# New protein deposition tracers in the pipeline

**DOI:** 10.1186/s41181-016-0015-3

**Published:** 2016-06-02

**Authors:** Aleksandar Jovalekic, Norman Koglin, Andre Mueller, Andrew W. Stephens

**Affiliations:** Piramal Imaging GmbH, Tegeler Straße 6-7, 13353 Berlin, Germany

**Keywords:** PET, Radiotracer, Beta-amyloid, Tau, Alpha-synuclein, Neurodegeneration

## Abstract

Traditional nuclear medicine ligands were designed to target cellular receptors or transporters with a binding pocket and a defined structure–activity relationship. More recently, tracers have been developed to target pathological protein aggregations, which have less well-defined structure–activity relationships. Aggregations of proteins such as tau, α-synuclein, and β-amyloid (Aβ) have been identified in neurodegenerative diseases, including Alzheimer’s disease (AD) and other dementias, and Parkinson’s disease (PD). Indeed, Aβ deposition is a hallmark of AD, and detection methods have evolved from coloured dyes to modern ^18^F-labelled positron emission tomography (PET) tracers. Such tracers are becoming increasingly established in routine clinical practice for evaluation of Aβ neuritic plaque density in the brains of adults who are being evaluated for AD and other causes of cognitive impairment. While similar in structure, there are key differences between the available compounds in terms of dosing/dosimetry, pharmacokinetics, and interpretation of visual reads. In the future, quantification of Aβ-PET may further improve its utility. Tracers are now being developed for evaluation of tau protein, which is associated with decreased cognitive function and neurodegenerative changes in AD, and is implicated in the pathogenesis of other neurodegenerative diseases. While no compound has yet been approved for tau imaging in clinical use, it is a very active area of research. Development of tau tracers comprises in-depth characterisation of existing radiotracers, clinical validation, a better understanding of uptake patterns, test-retest/dosimetry data, and neuropathological correlations with PET. Tau imaging may allow early, more accurate diagnosis, and monitoring of disease progression, in a range of conditions. Another marker for which imaging modalities are needed is α-synuclein, which has potential for conditions including PD and dementia with Lewy bodies. Efforts to develop a suitable tracer are ongoing, but are still in their infancy. In conclusion, several PET tracers for detection of pathological protein depositions are now available for clinical use, particularly PET tracers that bind to Aβ plaques. Tau-PET tracers are currently in clinical development, and α-synuclein protein deposition tracers are at early stage of research. These tracers will continue to change our understanding of complex disease processes.

## Introduction

Traditionally, nuclear medicine ligands were primarily designed for targeting cellular receptors or transporters. They were tightly bound, and often internalized or transported into the cell and trapped inside by metabolic transformation, while unbound ligand was cleared. More recently, a class of imaging tracers has become available whose members bind misfolded protein aggregates. This new paradigm requires different lead optimization, different types of analysis, and quantitation. Previous approaches targeted a binding pocket where derivatives of ligands displayed a defined structure-activity relationship. Examples of protein aggregate imaging include Aβ, tau, and α-synuclein. Such investigations required the design of a molecule that binds to β-sheets. The structure–activity relationship is less well defined, as no distinct binding pockets are present. Importantly, all protein depositions show a similar structural motif, and achievement of selectivity is the most important optimization goal. Nonetheless, protein sequence and aggregate structures are different enough that highly specific imaging agents have been developed for some of these targets.

**Fig. 1:**
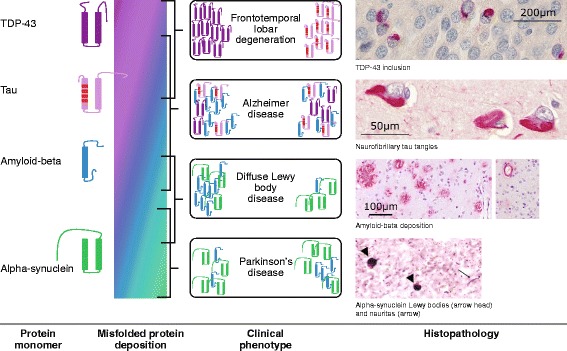
Schematic summary of key proteins present in frontotemporal lobar degeneration, Alzheimer’s disease, diffuse Lewy body disease and Parkinson disease. Protein monomers and their distribution for different clinical phenotypes are illustrated with symbolic drawings. Exemplary histopathology images are presented for TDP-43 inclusion, neurofibrillary tau tangles (immunohistochemistry with antibody AT8), amyloid-beta deposition (immunohistochemistry with monoclonal 6E10 Aβ antibody), and α-synuclein Lewy body inclusions (Images of TDP-43 inclusions, tau tangles, and Aβ deposits courtesy of Walter Schulz-Schaeffer, Goettingen, Germany)

Pathological protein depositions have been identified in a range of neurodegenerative diseases (Mollenhauer & Trenkwalder 2009). Tau and 43-kDa Tar DNA-binding protein (TDP-43) are present in neurofibrillary tangles characteristic of frontotemporal lobar degeneration and AD; Aβ plaques are the hallmark of AD; and α-synuclein has been identified in the pathognomonic bodies of diffuse Lewy body disease and PD. An overview of the misfolded protein depositions discussed in this article, with their associated histopathology and clinical manifestation, is presented in Fig. 1.

Co-pathologies have also been observed, in which more than one protein forms a deposition. Identification of in vivo biomarkers for such conditions will improve diagnosis and classification of patients, provide prognostic information, and improve the efficiency of drug development. This paper will discuss some of the new positron emission tomography (PET) tracers that are being developed to target misfolded protein depositions such as Aβ, tau, and α-synuclein.

## Review

### Established protein tracers–detection of Aβ plaques

AD is a chronic neurodegenerative disease that can now be detected in vivo by biomarkers years before clinical manifestation. The deposition of Aβ plaques is considered one hallmark in the pathogenesis of AD, and a hypothetical model of biomarker temporal evolution has been proposed that matches the sequence of molecular events proposed in the amyloid cascade hypothesis (Jack & Holtzman 2013). The model begins with Aβ42 overproduction and aggregation, with decreased clearance, followed by plaque formation. Thus Aβ-PET and cerebrospinal fluid (CSF) Aβ42 levels are the first markers to become abnormal in AD pathogenesis, although these biomarkers are not approved for prediction of disease progression or therapeutic monitoring.

The earliest methods of detecting Aβ plaques post mortem used coloured dyes–Congo red and thioflavin T–that bind to the β-sheet structure of Aβ (Glenner 1980). Thioflavin T was used as the basis for the development of the first radiolabelled molecules for use in PET. To date, several molecules have been studied in humans (Kung 2012): ^11^C-Pittsburgh compound B, ^18^F-florbetapir, ^18^F-florbetaben, ^18^F-flutemetamol, and NAV4694. (Table 1) ^18^F-florbetapir, ^18^F-florbetaben, and ^18^F-flutemetamol have been approved in Europe and US for clinical use in PET evaluation of Aβ neuritic plaque density in the brains of adults who are being evaluated for AD and other causes of cognitive impairment. While these agents are becoming increasingly established in routine clinical practice, there are important learnings from their clinical development and considerations that should be taken into account for future research and development of protein deposition tracers. Some of the peculiarities are described briefly in the following paragraphs.

**Table 1:** Overview Aß tracers

Tracer name	Chemical structure	Features
*Benzothiazole derivatives*		
[^11^C]-PiB (Klunk et al. 2004)		• Investigational
[^18^F]-flutemetamol (Vizamyl^TM^) (GE Healthcare 2014)		• Approved for clinical use
		• Injected dose: 185 MBq
		• Effective dose: 5.9 mSv (32 μSv/MBq)
		• Imaging window: 90-110 min p.i.
		• Scan duration: 20 min
		• Visual assessment: color
*Benzofuran derivative*		
[^18^F]-NAV4694 (formerly AZD4694) (Cselényi et al. 2012)		• Investigational
*Stilbene derivatives*		
[^18^F]-florbetaben (NeuraCeq^TM^) (Piramal Imaging 2014)		• Approved for clinical use
		• Injected dose: 300 MBq
		• Effective dose: 5.8 mSv (19 μSv/MBq)
		• Imaging window: 90-110 min p.i.
		• Scan duration: 20 min
		• Visual assessment: grey scale
[^18^F]-florbetapir (Amyvid^TM^) (Eli Lilly 2013)		• Approved for clinical use
		• Injected dose: 370 MBq
		• Effective dose: 7.0 mSv (19 μSv/MBq)
		• Imaging window: 30-50 min p.i.
		• Scan duration: 10 min
		• Visual assessment: grey scale

The three approved agents have a planar chemical structure that is suitable for binding to β-sheets in Aβ plaques. All approved agents follow the same mechanism of binding, but their different chemical structures lead to differences with regard to dosing and dosimetry; pharmacokinetics, including partitioning into grey and white matter structures; and interpretation of visual reads (Eli Lilly 2013; Piramal Imaging 2014; GE Healthcare 2014). For example, ^18^F-florbetapir and ^18^F-florbetaben PET images are approved for evaluation in greyscale, while ^18^F-flutemetamol PET images are read using a colour scale when used in the clinical setting. Thus each tracer requires a unique medical education programme to ensure reliable assessment of scans and to distinguish uptake in white matter from cortical grey matter.

Regulatory approval for Aβ PET scan assessment is currently based solely on a binary visual read-out, and all three reading methods have been validated against histopathology (Clark et al. 2012; Curtis et al. 2015; Sabri et al. 2015a). Of note, imaging agents used in oncology such as ^18^F-FDG or ^18^F-FLT become trapped in tumours leading to a stable or even increasing signal over time (Shields et al. 1998). In Aβ imaging, however, the tracer instead shows decreasing signal or standardised uptake values (SUVs) over time, as a result of washout after binding to Aß plaque-affected cortical areas. In addition, quantification of Aβ-PET scans typically involves calculating the SUV ratio, where the reference region is a region with a ligand uptake and washout pattern similar to Aß-plaque-affected cortical areas regardless of whether Aβ plaques are present (Schmidt et al. 2015). A number of different reference regions have been proposed (Landau et al. 2015), but further discussion is outside the scope of this review. Quantification of PET scans has the ability to better detect longitudinal changes during therapeutic intervention and has the potential for automated analysis via software with more detailed regional analysis. Future uses of Aβ-PET quantification, though not approved for routine clinical use, may include improved assessment in uncertain clinical cases, drug trial enrichment by patient selection, pre-symptomatic staging of disease, and therapeutic monitoring. Such uses require robust longitudinal assessment, reliable reference-region validation, and standardisation.

Beyond AD, amyloid-PET provides a unique opportunity for in vivo research of other conditions that are present with Aβ deposition. For example, Aβ-PET may also detect other plaque types and states of amyloid (e.g. diffuse plaques) (Sabri et al. 2015b), and thus may provide additional insights into the disease and its pathogenesis. Other conditions with Aβ-plaque depositions are reported, such as Lewy body diseases, cerebral amyloid angiopathy, brain trauma, and Down syndrome. As specific as the current tracers are for Aβ over other misfolded protein aggregates, somewhat surprisingly they do bind other amyloids outside the brain. ^18^F-Florbetaben and ^18^F-florbetapir have been reported to bind amyloid deposits in cardiac amyloidosis (Dorbala et al. 2014; Catafau & Bullich 2015; Mollee et al. 2015), and these tracers are also hypothesized to bind other peripheral amyloid deposits. In addition, tracers may also have value as a myelin biomarker in conditions such as multiple sclerosis (Matías-Guiu et al. 2015), by virtue of their white-matter signal.

### Protein deposition tracers under development

#### Detection of tau protein

Tau protein is the name given to soluble microtubule-associated protein (MAP), which is essential for regulating intracellular transport (Spillantini & Goedert 2013). Six different isoforms of tau exist, which can be distinguished by their number of binding domains (either three or four), and different forms are accumulated in different diseases (Delacourte 1999; Braak & Braak 1998). Furthermore, hyperphosphorylation and other post-translational modifications can have an impact on tau conformation, leading to, for example, aggregation in filamentous structures.

Tau protein aggregation leads to neuronal cell dysfunction and death, and studies show a strong association between tau deposits, decreased cognitive function, and neurodegenerative changes in AD. While the evolution of AD neuropathology depends on interactions between Aβ and tau (Jucker & Walker 2011), the relative contributions of the two proteins in the development of AD remain unclear. There is emerging evidence from studying hereditary Alzheimer’s Disease (e.g. DIAN study) that continues to point to a primary role of Aß in AD. Significant proportions of the observed variance in age at symptom onset can be explained by family history and mutation type (Ryman et al. 2014). Nevertheless, several other questions remain including the presence of Aβ deposition in cognitively normal individuals and time to development of first symptoms or the weak correlation between plaque load and cognition (Morris et al. 2014). Expanding the view of the AD pathogenesis beyond Aβ and tau pathology and considering aspects such as lifestyle, cognitive reserve may provide answers in the future. Imaging Aß and tau allows investigators to look at the impact on cognition and follow subjects from an earlier stage. In addition to AD several neurodegenerative diseases – including chronic traumatic encephalopathy, progressive supranuclear palsy, corticobasal degeneration, and some variants of frontotemporal lobar degeneration – have been described in which tau aggregate deposition is a dominant pathology (Mohorko & Bresjanac 2008; Lee et al. 2001; McKee et al. 2009).

Tau is a more complex target than Aβ in that the monomer protein is much larger than Aβ, is represented in different isoforms in different diseases, is present in lower amounts and has a distinct anatomic spread throughout the brain as the different diseases progress. These characteristics, and the intracellular localisation, make the requirements for a tau PET tracer more challenging (Villemagne et al. 2015).

Several tau imaging compounds have been described in preclinical and clinical studies. To date, however, none have been approved. The first ^18^F tracer with tau binding was ^18^F-FDDNP, although the compound suffered from a lack of selectivity (Kepe et al. 2013). Regional uptake patterns in the brain were therefore required to differentiate Aβ and tau. Meanwhile, more-selective tracers have become available. ^11^C-PBB3, allows tau imaging in AD and non-AD tauopathies such as corticobasal syndrome. However, the ^11^C label is not preferred, as it limits widespread use due to its short half-life (20 min) (Shimada et al. 2015). Studies with the ^18^F-labelled tracers THK-523 and THK-5117 showed that these compounds do not correlate with Aβ distribution, but instead follow the known distribution of tau (Harada et al. 2013). However, high retention in white matter limits their use in the clinical setting. An improved compound from the series, ^18^F-THK-5351, provided information on tau neurofibrillary tangle pathology in living individuals in initial studies (Harada et al. 2015). The usefulness for detection of tau pathology in pure tauopathies, however, needs to be demonstrated clinically. The Siemens (now Avid) compound ^18^F-T808 showed good preclinical properties as well as good pharmacokinetic characteristics in a first-in-human study, although development was hampered by strong defluorination (Chien et al. 2014). Another derivative, ^18^F-T807 (now AV1451), showed slower kinetics but good imaging data in AD as well as in some other tauopathies. Off-target activity in the striatum and choroid plexus is, however, described for this compound (Chien et al. 2013). In comparison with other compounds, ^18^F-T807 has been evaluated in the most subjects. Recently presented data on three Roche tau tracers in humans showed that ^18^F-RO6958948 has a promising clinical profile, with good brain uptake and little retention in cognitively normal young individuals (Wong et al. 2015). The agent also has a distribution broadly consistent with published post-mortem data, including low, homogenous uptake in controls, higher, heterogeneous uptake in AD, and a different binding pattern when compared with Aβ tracers. Notably there was no apparent brain penetration of radiolabelled metabolites and no defluorination. A clinical study is ongoing to collect test-retest and whole-body dosimetry data. Furthermore, first-in-human data of the Genentech tau tracer (^18^F-GTP1) were recently presented, indicating a promising clinical profile (Sanabria Bohorquez et al. 2015). Finally, ^18^F-PI-2014 was tested recently in humans and has shown uptake in tau-target regions consistent with tau binding (Piramal Imaging, data on file). Very recently, preclinical data from ^18^F-MK-6240 were published (Walji et al. 2016). This ^18^F-labeled agent combines good in vitro characteristics for NFT binding and clean off-target profile with suitable physicochemical properties and pharmacokinetics in rhesus monkeys. A clinical study is underway and results should be expected soon. A summary of tau tracer characteristics and key features of those with published structural information is presented in Table 2.

**Table 2:** Characteristics of published tau protein tracers updated from (Villemagne et al. 2015)

Tracer name	Chemical structure	Features
*Pyridinyl*-*butadienyl*-*benzothiazole derivative*		
[^11^C]-PBB3 (Maruyama et al. 2013; Hashimoto et al. 2014)		• Selectively binds to tau
		• Retention of ^11^C-PBB3 in the venous sinuses
		• Retention of tracer in basal ganglia in patient with corticobasal degeneration suggests that it might bind to non-AD tauopathies
*Dialkylamino*-*naphthylethylidene derivative*		
[^18^F] FDDNP (Kepe et al. 2013; Thompson et al. 2009; Small et al. 2013; Shoghi-Jadid et al. 2002; Smid et al. 2013)		• First ^18^F-tracer with tau binding
		• Lack of selectivity for tau; nanomolar binding affinity to Aβ
		• Very limited dynamic range
		• Regional brain retention used for differentiating Aβ and tau
*Benzimidazole derivatives*		
[^11^C]-N-Methyl-Lansoprazole (Shao et al. 2012; Fawaz et al. 2014)		• In vitro binding to paired helical filament-tau demonstrated
		• No brain uptake in mice (P-glycoprotein substrate)
		• Brain uptake in non-human primates
		• No human studies reported
[^18^F]-N-Methyl-Lansoprazole (Fawaz et al. 2014)		
*Quinoline derivatives*		
[^18^F]-THK-523 (Harada et al. 2013)		• Slow kinetics
		• Non-specific binding (white matter, brain stem)
		• No detection of non-AD tauopathies (Pick’s disease; three-repeat tauopathy)
[^18^F]-THK-5105 (Okamura et al. 2013; Okamura et al. 2014)		• Faster kinetics and higher contrast than ^18^F-THK-523
		• Non-specific binding (white matter, brain stem)
[^18^F]-THK-5117 (Okamura et al. 2013; Okamura et al. 2015)		
[^18^F]-THK-5351 (Harada et al. 2015)		• Faster kinetics and higher contrast than THK-523
		• Lower white matter retention
		• Higher signal-to-noise ratio compared with ^18^F-THK-5105 and ^18^F-THK-5117
*6*,*5*,*6 Tricyclic pyrimidines and indoles*		
[^18^F]-T807 (Chien et al. 2013; Xia et al. 2013)		• Tracer with broadest clinical data package
		• Cortical retention consistent with the known distribution of tau in AD brain
		• Strong correlation with disease severity
		• Slower kinetics than ^18^F-T808
		• Off-target activity (striatum, choroid plexus)
[^18^F]-T808 (Chien et al. 2014)		• Faster kinetics than ^18^F-T807
		• Substantial defluorination
*Pyrrolo*-*pyridine*-*isoquinolineamine*		
[^18^F]-MK-6240 (Walji et al. 2016)		• Good in vitro binding affinity to NFTs, high selectivity to β-amyloid, and excellent physicochemical properties for brain penetration and cellular permeability.
		• No off-target binding and suitable in vivo pharmacokinetics
		• Clinical studies are currently underway

*AD* alzheimer’s disease

Future development of tau tracers will require further evaluation of existing radiotracers, including preclinical characterisation, validation in the clinic, better understanding of uptake patterns in healthy controls, test-retest and human dosimetry data, and neuropathological correlations with PET, as well as head-to-head comparisons between different tracers. Improvement seems possible in the pharmacokinetic properties of ^18^F-labeled tracers, binding selectivity, and experience in non-AD tauopathies.

Overall, the combination of Aβ and tau-PET is currently significantly improving the knowledge of the interactions between the two proteins in humans. In addition, tau-PET–in its unique role as a marker of neurodegeneration–may allow the in vivo study of tau pathology evolution and topographic distribution across diseases. Tau imaging could also allow early, more accurate diagnosis, and more importantly monitoring of disease progression, in other tauopathies, cognitive impairment, movement disorders, and head trauma. Tau-PET may also lead to more efficient development of disease-modifying drugs not only for compounds targeting the tau protein itself.

### Detection of α-synuclein

Investigation of α-synuclein and TDP-43 in post-mortem human brains has led to increased understanding of the evolution of neuropathology in PD and amyotrophic lateral sclerosis, in which lesions are believed to spread from an initial ‘seed’ of misfolded protein (Jucker & Walker 2013). There is therefore a clinical need for imaging modalities for detection of α-synuclein, which has a potential role in the differential diagnosis of PD, dementia with Lewy bodies, progressive supranuclear palsy, and multiple system atrophy. Genetic biomarkers in these conditions, while critically important in the case of inherited disease, are not salient in the majority of cases (>90 %) with sporadic PD. Detection methods for α-synuclein in CSF are currently under development, although it is not clear how CSF levels relate to histopathology data (Mollenhauer 2014) and still need further validation.

Another role for α-synuclein imaging is to decrease risk and increase efficiency in drug discovery. Imaging could identify patients early enough for potential therapies, assist with therapeutic monitoring, and enhance trial recruitment and patient enrichment. α-synuclein has advantages over dopamine as a biomarker for PD, as changes in α-synuclein may occur earlier than dopamine changes, and are not up-or down-regulated by symptomatic treatment. Efforts to develop PET or single-photon emission computed tomography tracers for α-synuclein are ongoing but are still in their infancy. The compounds currently investigated for imaging α-synuclein depositions are shown in Table 3. Research groups started with the investigation of the ^18^F-labeled compound BF-227 that was reported to bind to both synthetic α-synuclein aggregates as well as β-amyloid fibrils in vitro (Fodero-Tavoletti et al. 2009). It was demonstrated that BF-227 could stain α-synuclein-containing glial cytoplasmic inclusions in post-mortem tissues. Moreover, a PET study with ^11^C-labelled BF-227 showed its ability to detect α-synuclein deposits in the living brains of patients with multiple system atrophy (Kikuchi et al. 2010). However, the high affinity of this radiotracer for β-amyloid plaques limit its use in humans for differential diagnosis.

**Table 3:** Characteristics of published a-synuclein deposition tracers

Tracer name	Chemical structure	Features
*Aminothiazolyl*-*ethenyl*-*benzoxazole derivatives*		
[^11^C] BF-227 (Kikuchi et al. 2010)		• Non-selective, affinities: see below for ^18^F-derivative
		• Investigated in MSA patients
[^18^F] BF-227: (Fodero-Tavoletti et al. 2009)		• Aß1-42 fibrils: K_D1_ = 1.3 nM
		• α-syn fibrils: K_D_ = 9.6 nM
*Phenothiazine derivatives*		
SIL23 (Bagchi et al. 2013)		• Affinity and selectivity not optimal for in vivo imaging
		• Affinity α-synuclein: K_i_ = 58 nM
		• Screening tool
[^18^F] 2b (Zhang et al. 2014)		• Affinity α-synuclein: K_i_ = 49 nM
		• Selectivity α -syn vs. Aß: 2-fold
		• Selectivity α -syn vs. tau: 2.5-fold
		• Crosses blood–brain-barrier in healthy cynomolgus macaques
		• Shows sufficient initial uptake and wash-out
		• Higher selectivity desired
[^11^C] 2a (Zhang et al. 2014)		• Affinity α-synuclein: K_i_ = 32 nM
		• Selectivity α-syn vs. Aß: 3-fold
		• Selectivity α-syn vs. tau: 4-fold
		• Crosses blood–brain-barrier in cynomolgus macaques
		• Shows sufficient initial uptake and wash-out
		• Higher selectivity desired
*3*-(*Benzylidene*) *indolin*-*2*-*one derivatives*		
[^18^F] 46a: (Chu et al. 2015)		• Selective for α-synuclein:
		o α-syn K_d_ = 8.9 nM
		o Aß K_d_ = 271 nM
		o Tau fibrils: 50 nM
		• High logP and presence of nitro group may limit its use for in vivo PET studies
		• Potential as secondary lead compound for further SAR studies

A series of phenothiazine derivatives was described for α-synuclein-binding (Yu et al. 2012) and the radioiodinated compound SIL23 was developed (Bagchi et al. 2013). As stated by its developers, the affinity of SIL23 for α-synuclein and its selectivity for α-synuclein versus Aβ and tau fibrils is not optimal for imaging fibrillar α-synuclein in vivo, but it could be used to screen additional ligands for suitable affinity and selectivity. Following this approach, additional compounds such as [^11^C] 2a and [^18^F] 2b have been identified that are more specific for α-synuclein and have shown the ability to cross the blood–brain barrier in animal studies (Zhang et al. 2014). However, these have not yet translated to human imaging. More recently, the same group reported the development and in vitro characterization of (benzylidene) indolin-2-one derivatives as new ligands for α-synuclein fibrils covering also PET ligands like [^18^F] 46a with high affinity and selectivity for α-synuclein (Chu et al. 2015). Future research will show whether some of these compounds have the ability to image α-synuclein depositions in patients.

## Conclusions

Several PET tracers for detection of pathological protein depositions or aggregates are now available for routine clinical use. In particular, PET agents binding to Aβ plaques are approved as an adjunct to other diagnostic evaluations to estimate the plaque density in patients with cognitive impairment who are being evaluated for AD or other causes of cognitive decline. Tau-PET tracers are currently in clinical development, and α-synuclein protein deposition tracers are at early stage of research. Importantly, PET tracer development and imaging of protein aggregates require different approaches to those involved in imaging of receptors or transporters, including lead optimisation, scan analyses and quantitation. These tracers have, and will continue to, change our understanding of complex disease processes.

## Abbreviations

Aβ: Beta-amyloid
AD: Alzheimer’s disease
CSF: Cerebrospinal fluid
PD: Parkinson’s disease
PET: Positron emission tomography
SUV: Standardized uptake value
TDP-43: 43 kDa Tar DNA-binding protein
Aβ42: Beta-amyloid beta (1–42)
